# Psychiatric Disorders after Switching to Dolutegravir: A Case Report of a 59-Year-Old Virosuppressed HIV-1 Positive Woman

**DOI:** 10.1155/2020/9708913

**Published:** 2020-04-14

**Authors:** Daniele Mengato, Raffaella Binazzi, Ivan Unterholzner, Elke M. Erne, Alicia Tavella

**Affiliations:** ^1^Department of Hospital Pharmacy, Bolzano Central Hospital, Bolzano, Italy; ^2^Infectious Diseases Unit, Bolzano Central Hospital, Bolzano, Italy; ^3^Department of Internal Medicine, Silandro Hospital, Silandro, Italy

## Abstract

We report a case of a woman who experienced psychiatric disorders after switching her antiretroviral therapy (c-ART) to dolutegravir (DTG). She is a 59-year-old HIV-1 positive woman with a recent story of cardiovascular disorders treated with beta-blockers, clopidogrel, and rosuvastatin. She underwent a c-ART switch from darunavir/cobicistat and maraviroc to emtricitabine/tenofovir alafenamide fumarate in association with dolutegravir due to drug-drug interactions. One week later, she started to show psychiatric symptoms that required admission to the psychiatric unit. These disorders resolved within a couple of days after DTG discontinuation to allow a regular discharge. With this case-report, we would like to analyse the possible correlation between integrase inhibitor and severe psychiatric disorders in order to confirm the evidences already published in literature.

## 1. Introduction

Since 2014, right after its entry in clinical practice, dolutegravir showed its efficacy and safety in HIV-1 positive patients. It is the third integrase inhibitor available after raltegravir and elvitegravir, the first one requiring a once-daily intake [1, 2].

Numerous cohort and randomized clinical trials demonstrated the excellent safety profile and antiviral activity of this pharmacological class. Dolutegravir, in particular, is characterized by a good tolerability and high genetic barrier [1].

## 2. Case Presentation

We report a case of a woman who experienced psychiatric disorders after switching to dolutegravir.

She is a 59-year-old HIV-1 positive woman with a recent diagnosis of transitory ischemic attack (TIA) started on beta-blockers, clopidogrel, and rosuvastatin. She was switched from darunavir/cobicistat and maraviroc 150 mg/BID to emtricitabine/tenofovir alafenamide fumarate 200 mg/25 mg in association with dolutegravir 50 mg once daily due to drug-drug interactions. She started dolutegravir on April 13, 2018, and right after the first three doses, she started to show psychiatric symptoms such as anxiety, depression, and irritability.

One week later, she experienced symptoms' worsening that required admission to the psychiatric unit.

Dolutegravir was stopped immediately, and treatment was switched to maraviroc 150 mg BID again. Psychiatric disorders resolved within a couple of days after DTG discontinuation to allow a regular discharge.

The patient and her stakeholders did not report any history of psychiatric troubles and/or antipsychotic or antidepressive drugs' use, apart from an adverse event occurring after an antibiotic treatment with rifampicin, resolved immediately after drug's discontinuation. Other drugs did not present any drug-drug interactions with the c-ART. Patient reports no past use of psychoactive substances or drugs.

The case was reported regularly to the local pharmacovigilance center.

## 3. Discussion

To the best of our knowledge, this is the second case report describing psychiatric disorders possibly correlated with dolutegravir. In 2015, Kheloufi et al. described a case series of four patients who experienced different psychiatric disorders after switching to dolutegravir [3].

The main difference in our finding is correlated with the timing of the onset of the disorders. Kheloufi et al. reported that symptoms started to appear after few days of treatment, but discontinuation of therapy was performed usually at least a month later. In our case, we had to stop the therapy immediately due to the severity of the symptoms. The patient rapidly improved her psychiatric condition, permitting an early discharge.

Although common psychiatric adverse effects such as abnormal dreams, insomnia, anxiety, and depression are mentioned in the summary of product characteristics of dolutegravir, no study described such severe symptoms as we described [2].

Several postmarketing studies, however, described an exacerbation of preexisting psychiatric troubles or depression in patients treated with elvitegravir and raltegravir, the other two drugs of the integrase inhibitors class. These observations might suggest the hypothesis of a class effect, confirming the necessity to analyse in deep the psychiatric history of the patients before starting a therapy including one of these drugs [4, 5].

Our patient reported similar ADRs after an antibiotic therapy with rifampicin. It is well known that drugs metabolised by glucuronidation, in particular by UGT1A1, could lead to toxicity due to the presence of several enzyme mutations [6, 7]. A correlation between CNS' ADRs and the presence of UGT1A1 mutations should be investigated whenever to confirm or deny this evidence. Yagura et al. suggested a relationship between specific UGT1A1 polymorphism—UGT1A1^*∗*^6 and ^*∗*^28—and high trough concentrations of DTG, resulting in an increased risk of developing CNS' ADRs [8]. Such relationship between UGT1A1 polymorphism and drug metabolism was previously shown by other studies like the occurrence of irinotecan-associated toxicity and the presence of UGT1A1 gene polymorphism in the treatment of metastatic colorectal cancer [9]. No data about the presence and rate of UGT1A1^*∗*^6 and ^*∗*^8 polymorphism in the Italian and Austrian population are so far available.

Besides, being DTG coadministered with other HIV-drugs, an additional role in the disposition of DTG could be played by drug transporters. DTG is a substrate for Pgp and BCRP, two important drug transporters involved in the intestinal absorption and the CNS penetration of many HIV and non-HIV drugs [10].

We reported, finally, that even a patient without a known history of psychiatric troubles could be exposed to a risk with dolutegravir, probably due to the great ability to diffuse into the CNS.
